# Genomic characterization of antimicrobial resistance and mobile genetic elements in swine gut bacteria isolated from a Canadian research farm

**DOI:** 10.1186/s42523-025-00432-w

**Published:** 2025-06-18

**Authors:** Nahidur Rahman, Taylor McCullough, Daniel Flores Orozco, Sean Walkowiak, Abdolvahab Farzan, Shahrokh Shekarriz, Michael G. Surette, Nazim Cicek, Hooman Derakhshani

**Affiliations:** 1 Department of Animal Sciences, Faculty of Agricultural and Food Sciences, University of Manitoba, Winnipeg, MB Canada; 2 Grain Research Laboratory, Canadian Grain Commission, Winnipeg, MB Canada; 3 Ontario Veterinary College, University of Guelph, Guelph, ON Canada; 4 Farncombe Family Digestive Health Research Institute, McMaster University, Hamilton, ON Canada; 5 Department of Medicine, McMaster University, Hamilton, ON Canada; 6 Department of Biosystems Engineering, Faculty of Agricultural and Food Sciences, University of Manitoba, Winnipeg, MB Canada

**Keywords:** Swine gut microbiome, Antimicrobial resistance, Mobile genetic elements, Integrative and conjugative elements

## Abstract

**Introduction:**

The widespread use of antimicrobials in the livestock industry has raised global concerns regarding the emergence and spread of antimicrobial resistance genes (ARGs). Comprehensive databases of ARGs specific to different farm animal species can greatly improve the surveillance of ARGs within the agri-food sector and beyond. In particular, defining the association of ARGs with mobile genetic elements (MGEs)—the primary agents responsible for the spread and acquisition of resistant phenotypes among bacterial populations—could help assess the transmissibility potential of clinically relevant ARGs. Recognizing the gut microbiota as a vast reservoir of ARGs, we aimed to generate a representative isolate collection and genome database of the swine gut microbiome, enabling high-resolution characterization of ARGs in relation to bacterial host range and their association with MGEs.

**Results:**

We generated a biobank of bacteria from different sections of the gastrointestinal tracts of four clinically healthy pigs housed at a research farm in Ontario, Canada. The culturing was performed under anaerobic conditions using both selective and general enrichment media to ensure the capture of a diverse range of bacterial families within the swine gut microbiota. We sequenced the genomes of 129 unique isolates encompassing 44 genera and 25 distinct families of the swine gut microbiome. Approximately 85.3% (110 isolates) contained one or more ARGs, with a total of 246 ARGs identified across 38 resistance gene families. Tetracycline and macrolide resistance genes were the most prevalent across different lineages of the swine gut microbiota. Additionally, we observed a wide range of MGEs, including integrative conjugative elements, plasmids, and phages, frequently associated with ARGs, indicating that the swine gut ecosystem is conducive to the horizontal transfer of ARGs. High-throughput alignment of the identified ARG-MGE complexes to large-scale metagenomics datasets of the swine gut microbiome suggests the presence of highly prevalent and conserved resistome sequences across diverse pig populations.

**Conclusion:**

Our findings reveal a highly diverse and relatively conserved reservoir of ARGs and MGEs within the gut microbiome of pigs. A deeper understanding of the microbial host range and potential transmissibility of prevalent ARGs in the swine microbiome can inform development of targeted antimicrobial resistance surveillance and disease control programs.

**Supplementary Information:**

The online version contains supplementary material available at 10.1186/s42523-025-00432-w.

## Introduction

Swine farms are among the most significant contributors to the antimicrobial resistance (AMR) crisis within the agri-food sector. With a global pig population of approximately 1 billion in 2022 [1], the swine industry accounts for over 32% of total meat consumption worldwide and remains a major consumer of antimicrobials [2]. Large quantities of orally administered agents like tetracyclines, penicillin, and macrolides are still widely used in swine production, with the industry projected to drive 45% of the anticipated 11.5% increase in antimicrobial use in food-producing animals by 2030 [3]. The use of antimicrobials exerts selective pressure that favors the proliferation of resistant microbes, leading to the emergence and spread of antimicrobial resistant genes (ARGs) within and between microbial populations [4, 5]. The gut microbiome, with its high microbial density, is an ideal environment for the emergence and dissemination of ARGs [6]. Recent metagenomic studies have revealed an extensive resistance repertoire in the gut microbiomes of humans and animals, including resistance to medically important antimicrobials [7–11]. Opportunistic pathogens such as Enterococci have been shown to acquire or transfer their ARGs to other pathogens [12]. Such dissemination can occur through horizontal gene transfer (HGT), a process by which a microbe can obtain genes from a species outside its own lineage [13].

Self-transmissible mobile genetic elements (MGEs), including conjugative plasmids, bacteriophages, and integrative conjugative elements (ICEs) are the primary agents responsible for the horizontal transfer of genes among bacterial genomes [14]. Traditionally, these MGEs have been studied in the context of pathogenicity and transfer of virulence genes among clinical pathogens [14]. However, as the number of publicly available genome assemblies increases for isolates from the human and animal microbiota, it is becoming increasingly evident that the prevalence and diversity of MGEs associated with ARGs in commensal bacteria are at a scale comparable to pathogens [15]. In particular, ICEs play a significant role in inter-species gene transfer within the microbiomes of humans and animals, carrying genes linked to bacterial defense and metabolism [14, 16]. So, understanding the diversity and structure of MGEs in livestock microbiomes is critical for assessing their overlap with ARG reservoirs in zoonotic pathogens.

In recent years, assembly-based metagenomic approaches have provided valuable insights into the composition and functions of gut microbiome [17–19]. However, there are inherent limitations with the accuracy of metagenome-assembled genomes (MAGs) as these assemblies are often more fragmented than isolate genome assemblies, caused in part due to differential coverage of inter- and intra-species identical genomic regions and repeat elements, resulting in mis-assemblies and chimerism [20]. Also, MAGs frequently fail to capture the full spectrum of core and variable genes in diverse microbial populations [21]. Integrating culture-based approaches with whole genome sequencing (WGS) enables the isolation of individual strains, the generation of high-quality genomes, and the contextualization of ARGs and MGEs within their specific hosts. Additionally, it facilitates phenotypic studies, such as antimicrobial susceptibility testing (AST), which are critical for linking genetic data to functional traits.

Given the significant AMR reservoir harbored within the gut microbiome of livestock [18, 22], the development of high-quality genome database of gut isolates can serve as a valuable resource for tracking the evolution, diversity, and distribution of ARGs within the agri-food sector. In this study, we established a collection of gut isolates from various sections of the gastrointestinal tract of clinically healthy pigs with no prior exposure to antimicrobials. Our objectives were to identify the most prevalent classes of ARGs across distinct gut microbiota lineages and evaluate the correspondence between genotypic and phenotypic AMR within a subset of commensal gut isolates. Furthermore, we employed a non-reference-based approach to examine the prevalence of MGEs associated with different ARG classes and explore the distribution of the identified ARG-MGE complexes across diverse swine populations.

## Methods

### Media and culture conditions for isolation of swine isolates

The swine fecal and tissue samples used in this study were collected as part of a larger observational study on Canadian swine farms at the University of Guelph (Guelph, ON, Canada), the animal use was approved by the University of Guelph Animal Care Committee (AUP# 3124) and follows Canadian Council of Animal Care guidelines (CCAC, 2009). In brief, fresh samples were collected from clinically healthy animals, including two sows and two piglets, with no gastrointestinal symptoms and no history of antimicrobial therapy within 3 months of collection. Immediately after collection, samples were transferred to a sterile container and stored in sealed bags containing an anaerobic pouch (GasPak™ EZ; BD, MD, USA) and icepack. Samples were transferred to the laboratory within 3 h of collection and were further processed in an anaerobic chamber (5% CO_2_, 5% H_2_, 90% N_2_; Shel Labs, OR, USA). The following media were used for general enrichment and isolation of gut isolates: (a) BHIS: brain heart infusion agar (BD, NJ, USA) supplemented with 0.5 g/L L-cysteine hydrochloride hydrate, 10 mg/L hemin, and 1 mg/L vitamin K, (b) Cooked meat broth + 1.5% (w/v) agar, (c) Fastidious anaerobe agar (Neogen, MI, USA), and (d) Gifu anaerobic medium (Himedia Laboratories, Mumbai, India). We also used phenylethyl alcohol agar which is selective for gram-positive bacteria (ThermoFisher Scientific, Mississauga, ON, Canada). Individual colonies were picked, re-streaked to purity, and classified via PCR amplification of the V3 region of the 16S rRNA gene, following established protocols [23]. Sequencing was conducted on an Illumina MiSeq platform at McMaster University’s genomic facility. Isolates with non-redundant 16S rRNA genes clustered at 97% identity threshold were selected for whole genome sequencing.

### Genome sequencing and taxonomic classification of isolates

Genomic DNA was extracted from cell pellets using the Wizard Genomic DNA Purification Kit (Promega, WI, USA). DNA concentrations were quantified by Qubit dsDNA HS kit (ThermoFisher Scientific, Mississauga, ON, Canada). Illumina libraries were prepared according to a miniaturized library preparation protocol as previously described [24], using the NEBNext Ultra II FS DNA Library Prep Kit (NEB, MA, USA). Libraries were subjected to dual size-selection using the ProNex^®^ Size-Selective Purification System (Promega, WI, USA) to enrich for insert sizes of 800–1000 bp. Final libraries were sequenced on an Illumina HiSeq2500 platform in rapid run mode, paired-end 2 × 250 nt, at the McMaster Metagenomics Facility (Hamilton, ON, Canada). Trimmomatic (v.0.39) was used for quality trimming and removal of adapters from Illumina reads [25] followed by de novo assembly of genomes using Unicycler (v.0.4.8; default parameters, normal bridging mode) [26]. Resulting genomes were dereplicated using the dRep package (v.3.0.0; https://github.com/MrOlm/drep) at 99% average nucleotide identity (ANI) and included in downstream data analyses pipeline. The default parameters of the classify workflow of the Genome Taxonomy Database Toolkit (GTDB-TK v2.1.0) [27] were used for taxonomic classification and construction of phylogenetic tree based on multiple sequencing alignment of 120 ubiquitous bacterial single-copy genes. The online Interactive Tree of Life (iTol; v.6.0) toolkit was used for visualization of phylogenetic trees [28].

### Identification of antimicrobial resistance genes (ARGs) and mobile genetic elements (MGEs)

Genomes were screened for the presence of ARGs using the Resistance Gene Identifier tool (RGI v5.1.0) and the Comprehensive Antibiotic Resistance Database (CARD v3.1.4) [29], retaining only “strict” and “perfect” hits flagged by RGI. Additionally, the NCBI AMRFinderPlus toolkit (v.4.0.15) [30] was also used to verify the presence of ARGs identified by CARD-RGI, and only those ARGs identified by both toolkits were retained for downstream analyses. Next, the size of ARG-containing contigs were calculated, contigs smaller than 2 kbp were removed from downstream analyses, and bedtools (v2.30.0) [31] was used to retrieve up to 40 kbp upstream and downstream flanking regions of each ARG. A flanking length of 40 kbp was chosen to maximize the likelihood of identifying large chromosomal MGEs, including ICEs, which exhibit considerable size variation, ranging from as small as 20 kbp to over 500 kbp [32, 33]. Virsorter2 (v2.2.3) [34], PHASTER (online version accessed June 2022) [35], and PLSDB (v2020.11.19) [36] were used to identify potential bacteriophage and plasmid related sequences in the flanking regions of ARGs. Criteria for considering viral sequences using Virsorter2 included “max_score > 0.70; viral hallmark gene count > 1; and contig size > 10 kbp”. In addition to searching for known plasmid sequences in the PLSDB database, we also used the BAKTA toolkit (v1.10.4) [37] for comprehensive annotation of the flanking regions of ARGs to identify potential plasmid replication protein sequences (*repA* and *repB*). Further, to identify different components of ICEs, a unified profile hidden Markov model (pHMM) was compiled by concatenating individual pHMMs belonging to relaxase (PF03432), resolvase (PF00239), recombinase (PF07508), and phage-like integrase (PF00589) protein families, all obtained from the Pfam database [38], and the type IV secretion system (T4SS) pHMM of the CONJscan [39]. Flanking regions of ARGs were then classified into 4 categories: (A) ICEs: those containing an integration-related gene (i.e., site-specific recombinase, resolvase, and/or integrase), and three or more essential components of the conjugation system including relaxases, type IV coupling protein (T4CP), and any other components of a type IV conjugation system (i.e., VirB4 and VirB11 ATPases, and other proteins of the mating pair formation system and substrate translocation channel) [40], (B) Putative ICEs: those containing an integration-related gene and at least one conjugation related gene, (C) Integrative mobile elements (IMEs): those containing integration-related gene and relaxase, but lacking genes related to conjugation system, and (D) Putative MGEs: those lacking integration-related genes but containing at least one conjugation-related gene. Further, due to underrepresentation of extrachromosomal MGEs across draft genome assemblies, we performed specialized plasmid assembly on the raw sequencing reads using the Spades assembly toolkit (v4.1.0; using the default*–plasmid* parameter) [41]. The resulting contigs were screened by the ViralVerify tool (https://github.com/ablab/viralVerify) for identification of circular replicons of potential viral or plasmid origin. The resulting circular contigs were further annotated by Virsorter2 (v2.2.3) for identification of putative viral and searched against the PLSDB plasmid database for identification of putative plasmids [42]. We also used the BAKTA toolkit (v1.10.4) for comprehensive annotation of the circular contigs, particularly for identification of replication protein sequences (*repA* and *repB*) and mobilization (*MOB*) proteins. Moreover, the combination of RGI and NCBI AMRFinderPlus toolkits were also used for identification of ARGs in the identified circular contigs.

### Assessing antimicrobial susceptibility phenotype for selected ARGs

We selected eleven antimicrobials, including tetracycline, clindamycin, gentamicin, ciprofloxacin, erythromycin, ampicillin, vancomycin, rifampicin, metronidazole, trimethoprim, and chloramphenicol, each representing a different antimicrobial class, based on genomic analysis, for performing antimicrobial sensitivity test using disc diffusion assays (Oxoid, Basingstoke, UK). For the initial resistance screening, we tested 20 isolates that carried resistant genes against various antimicrobial classes, as identified through genomic analysis. The optical density (OD 600 nm) of cultures was adjusted to obtain cell density equivalent to 0.5 McFarland scale (cell count of ~ 10^8^ CFU ml^−1^). Isolates were then inoculated on BHI agar inside anaerobic chamber with corresponding antimicrobial discs and incubated at 37 °C for 24 h. The following day, based on the results of the disc diffusion tests, we selected nine isolates for validation of resistant phenotype using E-test strips (Liofilchem strips, Italy) to further determine the minimum inhibitory concentrations (MICs) for these isolates (0.5 McFarland standard CFU). Isolates were inoculated on BHI agar with E-test strips. After incubating at 37 °C for 24 h, MICs of isolates were recorded. Isolates were considered resistant based on the clinical breakpoints reported by the European Committee on Antimicrobial Susceptibility Testing (EUCAST) [43]. For cases where breakpoints were unavailable, the MIC values reported by Bengtsson-Palme et al. [44] were used.

### Assessing the distribution of the ARG-associated MGEs in other pig populations

Finally, to further assess the presence and distribution of homologous sequences to the identified ARG-associated MGEs in the gut microbiome of other swine populations, metagenomic sequencing reads representing the fecal microbiome of 287 pigs from France (n = 100), Denmark (n = 100) and China (n = 87) (available through the European Nucleotide Archive (ENA) under accession code PRJEB11755 [45]) were mapped against the retrieved fraction of contigs containing ARGs and signature genes of MGEs, as well as those circular contigs of potential viral or plasmid origins. Raw reads were mapped using BWA-MEM (v.0.7.17; default parameters) and sorted using SAMtools view (v.1.17). The number of mapped reads was identified through SAMtools coverage (v.1.17). Samples were considered to contain the identified ARG-associated MGEs if their metagenomes had greater than 80% coverage of each ARG-MGE sequence (or more than 20,000 bp coverage for long contigs) with a minimum of 10 × average base coverage across the aligned region.

## Results

### Biobank and genome database of the swine gut isolates

**Fig. 1:**
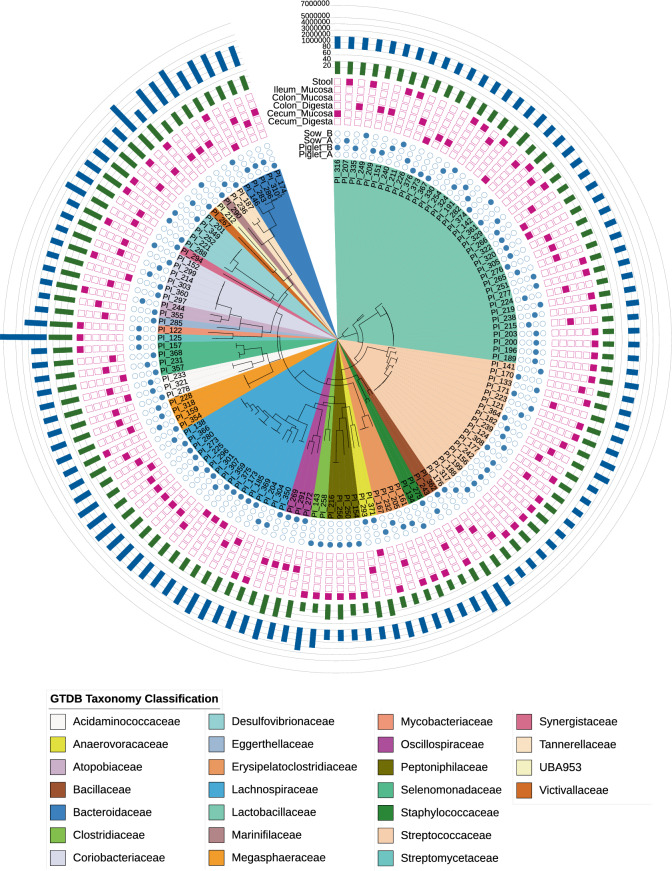
Phylogenetic diversity of swine isolate collection. Whole genome sequencing was performed on 129 unique isolates recovered from four healthy pigs. Phylogenetic relatedness among different species was measured by constructing a maximum-likelihood tree using 120 bacterial marker genes of the GTDB-Tk and visualized using the software (https://itol.embl.de). Microbial clades on the tree are coloured according to the taxonomic classification at the family level. The outer rings from inside to outside represent host animal, sample source, GC content, and genome size of each isolate

To evaluate the diversity and distribution of ARGs and their associated MGEs in the swine gut microbiome, we performed WGS on 129 unique isolates recovered from four clinically healthy pigs at different ages. Of these, 53 isolates were cultured from the stool of two adult sows and 76 isolates from digesta and mucosa of colon, cecum, and ileum of two piglets. The resulting database encompasses 25 different families, 44 genera, and 50 species of bacteria (Fig. 1). The most prevalent families were *Lachnospiraceae* (sixteen isolates belonging to 8 distinct genera including members of *Bariatricus, Blautia, Coprococcus, Fusicatenibacter, Lachnoclostridium, Mediterraneibacter, and Sporofaciens*), *Lactobacillaceae* (thirty-seven isolates belonging to *Lactobacillus, Ligilactobacillus,* and *Limosilactobacillus* genera), and *Streptococcaceae* (eighteen isolates within the *Streptococcus* genus). Other families of note include *Coriobacteriaceae* (six isolates within the *Collinsella* genus), *Bacteroidaceae* (five isolates within the *Prevotella* genus), and *Desulfovibrionaceae* (five isolates within the *Desulfovibrio* genus). Genome sizes varied from 1656 to 7667 kbp, with an average genome size of 2513 kbp. Typical of bacterial genomes, a wide range of GC content was observed, from as low as 27.84% in one *Clostridium spp.* isolate to as high as 73.12% in a *Streptomyces albidoflavus* isolate. The average genome completeness and contamination across isolates were 99.08% and 0.58%, respectively (Fig. 1, and Additional File 1).

### Distribution of antimicrobial resistance genes

**Fig. 2:**
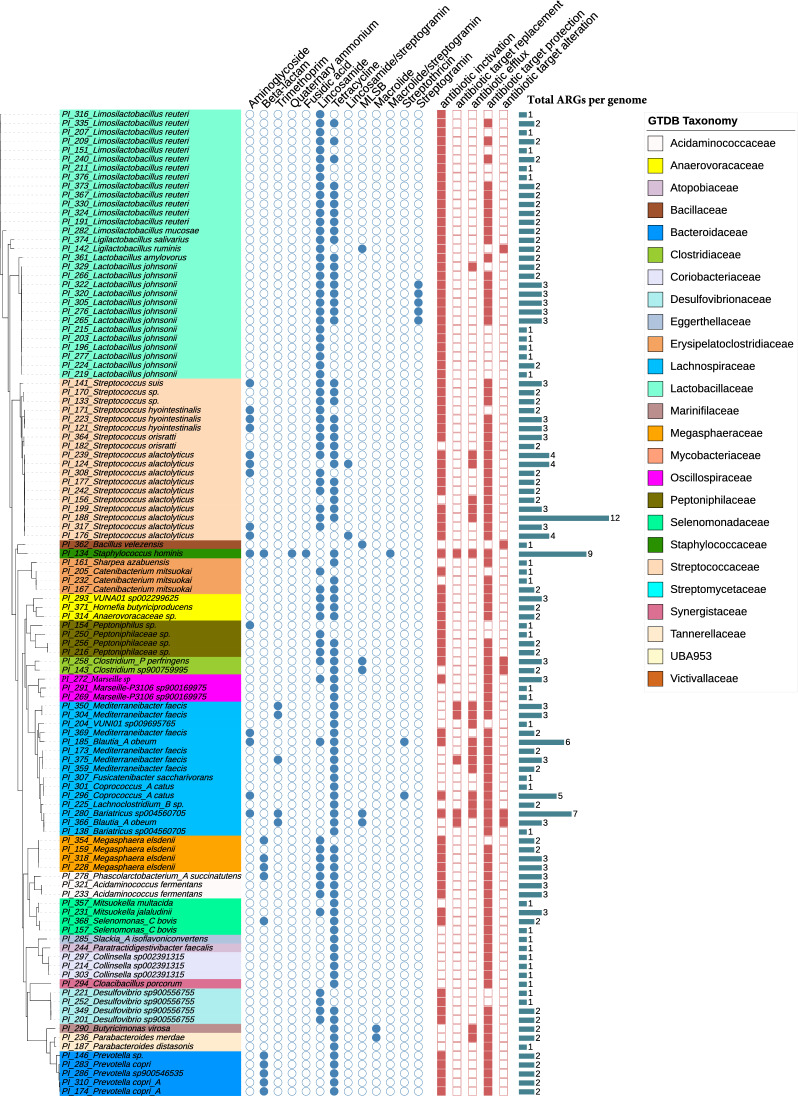
Distribution of ARGs across microbial species. The ARGs were identified using the Resistance Gene Identifier (RGI) from the CARD database and validated by NCBI AMRFinderPlus. The ARGs were categorized based on the mechanisms of resistance and the specific antimicrobials to which they confer resistance. Bar charts show the total number of ARGs detected in each species. Microbial clades on the tree are coloured according to the taxonomic classification at the family level

Genes conferring resistance to antimicrobials were widespread across host-associated microbiota in our study. Of the 129 isolate genomes, 85.3% (110 isolates) contained one or more ARGs, which included 42 isolates from sow stool, 48 from piglet cecum and colon digesta, and 20 from piglet tissue. In total, 246 predicted ARGs were identified across 38 resistance gene families, including 93 ARGs from isolates originating from sow stool, 41 from piglets’ mucosa isolates, and 112 from piglets’ digesta isolates (Additional File 2). These ARGs confer resistance to a range of antimicrobial drugs relevant to human and animal medicine, including aminoglycosides (e.g., *ANT(6)-Ia*, *APH(3')-IIIa)*, beta-lactams (e.g., *ACI-1)*, and tetracyclines (e.g., *tetWNW*, *tetQ, tetW).* The distribution of ARGs among the isolates is shown in Fig. 2.

Tetracycline resistance genes were the most abundant, with *tetWNW* exhibiting the broadest host range, detected in 46 isolates across 13 families, including *Lactobacillaceae* (n = 16), *Streptococcaceae* (n = 6), and *Acidaminococcaceae* (n = 5). *TetO* was found in 15 isolates with a narrower distribution, primarily within *Lachnospiraceae* (n = 7). 41 of the contigs carrying tetracycline resistance genes were identified in sow fecal isolates, while 55 were found in piglet digesta, and 15 in piglet mucosal isolates. Lincosamide resistance genes were also prevalent, with *lnuC* detected in several families, including *Lactobacillaceae* (n = 14), *Megasphaeraceae* (n = 4), and *Peptoniphilaceae* (n = 4).

The most prevalent mechanisms of resistance among the identified ARGs were antibiotic inactivation, target protection, and efflux pumps. A total of 110 ARGs encoding antibiotic inactivation mechanisms were detected across 14 bacterial families, including *Lactobacillaceae* (n = 36) and *Streptococcaceae* (n = 27). A total of 99 ARGs encoding antibiotic target protection were detected in 21 families, including *Streptococcaceae* (n = 23) and *Lactobacillaceae* (17). Efflux pumps were associated with 23 ARGs across 11 families, including *Lactobacillaceae* (n = 10) and *Streptococcaceae* (n = 8). Less common mechanisms included target replacement (n = 8) in *Lachnospiraceae* (n = 5), and *Staphylococcaceae* (n = 3), and target alteration (n = 6) in *Clostridiaceae* (n = 2) and *Lachnospiraceae* (n = 2) (Fig. 2 and Additional File 1).

Most isolates carried multiple resistance genes, with 77 of 129 isolates (59.7%) containing two or more ARGs (Fig. 2). For example, *Streptococcus alactolyticus* (PI_188) harbored ARGs for lincosamide (*lnuA*) and tetracycline (*tet45*, *tetWNW*). *Streptococcus suis* (PI_141), a potential pig pathogen, contained threeARGs conferring resistance to aminoglycosides (*ANT(6)*), lincosamides (*lnuB*), and tetracycline (*tetM*). *Staphylococcus hominis* (PI_134) had nine ARGs linked to resistance against aminoglycosides (*ANT(4)*), fluoroquinolones (*qacA*), fusidic acid (*fusB*), macrolides/streptogramins (*msrA*), methicillin (*mecA*, *mecI*, *mecR1*), tetracycline (*tetK*), and beta-lactamase (*blaZ*).

In addition to opportunistic pathogens, isolates identified as gut commensals also carried multiple ARGs. For example, three *Lachnospiraceae* isolates harbored at least five ARGs. *Lachnoclostridium_B* (PI_185) contained genes for tetracycline (*tet40*, *tetO*), aminoglycoside (*aad(6)*, *APH(3)*), lincosamide (*lnuC*), and streptothricin (*SAT-4*). A *Bariatricus* isolate (PI_280) carried seven ARGs, including resistance to aminoglycosides (*ad(6)*, *APH(3)*), lincosamides (*ErmG*), and tetracyclines (*tet40, tetWNW*). Similarly, *Coprococcus catus* (PI_296) harbored ARGs for aminoglycosides (*aad(6)*, *APH(3)*), streptothricin (*SAT-4*), and tetracycline (*tet40*, *tetO*).

### Discrepancy between antimicrobial susceptibility genotype and phenotype

Antimicrobial susceptibility tests (AST) were conducted to assess antimicrobial resistance phenotypes in various isolates harboring ARGs (Supplementary File 2). The results revealed that some isolates with tetracycline resistance genotype, such as *Streptococcus alactolyticus* (isolates PI_188, PI_239) and *Staphylococcus hominis* (PI_134), exhibited the resistance phenotype. Similarly, *Coprococcus catus* (PI_296) and *S. alactolyticus* (PI_239) displayed resistance to aminoglycosides having ARGs for this antimicrobial. However, there were few instances where isolates showed resistance to certain antimicrobials without possessing ARGs. For example, *S. hominis* (PI_134) and *C. catus* (PI_296) were resistant to metronidazole, and both *Mitsuokella jalaludinii* (PI_231) and *C. catus* (PI_296) were resistant to trimethoprim, even though no resistance genes for these antimicrobials were detected in their genomes. On the other hand, there were several isolates where no resistance phenotype was observed in the AST, even though the isolates carried the corresponding ARGs (Additional File 3).

### Association of ARGs with mobile genetic element (MGEs)

**Fig. 3:**
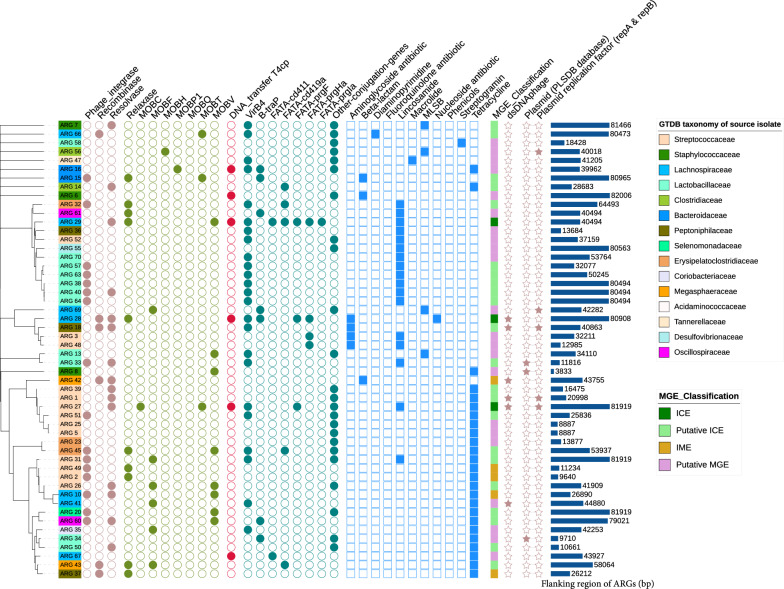
Classification of ARG-associated MGEs: The tree was created by assessing Mash distances between the flanking regions of each ARG (40 kbp upstream and downstream) using MashTree (https://github.com/lskatz/mashtree). The color of each node corresponds to the phylogenetic lineage of the host species. Signature mobility genes were identified using profile hidden Markov models (pHMM) of relaxase (PF03432), resolvase (PF00239), recombinase (PF07508), phage-like integrase (PF00589), and the type IV secretion system (T4SS obtained from CONJscan). The flanking regions were categorized as Integrated Conjugative Elements (ICEs), Putative ICEs, Integrative Mobile Elements (IMEs), and other putative conjugative MGEs based on the profile of mobility genes. Virsorter2 (v2.2.3), PHASTER, and PLSDB (v2020.11.19) were utilized to detect potential bacteriophage and plasmid-related sequences in the flanking regions of ARGs. The bar chart illustrates the length of sequences obtained from the flanking regions of ARGs, with regions shorter than 80 kbp indicating overlap with contig ends

We screened 40 kbp upstream and downstream of each ARG for MGE signature genes. A total of 55 ARGs were found to be linked to MGEs, including plasmids, phages, ICEs, and IMEs (Fig. 3). Three plasmid-associated ARGs were detected using the search engine of the PLSDB database, including *lnuA* (lincosamide resistance) in *Limosilactobacillus reuteri* and tetracycline resistance genes (*tetK*, *tetWNW*) in *Staphylococcus hominis* and *L. reuteri*. Additionally, our reference-free search for homologous sequences to plasmid replication proteins (repA and repB) also identified five ARGs with potential plasmid-related genes in their 40kbp flanking regions, including those carrying MLSB resistance in *Lachnospiraceae* and *Clostridiaceae* species, aminoglycoside resistance in *Lachnospiraceae* and *Peptoniphilaceae* species, and tetracycline resistance in two *Streptococcaceae* species. Eight ARGs were linked to dsDNA phages, three of which conferred tetracycline resistance in *Streptococcaceae* and *Lachnospiraceae* isolates. Two phage-associated ARGs exhibited multidrug resistance, including aminoglycoside, lincosamide, nucleoside, and tetracycline resistance, in *Streptococcaceae* and *Lachnospiraceae*. Two additional phage-associated ARGs conferred beta-lactam and aminoglycoside resistance in *Megasphaeraceae* and *Peptoniphilaceae* isolates, respectively.

Using profile HMMs, we identified signature genes of mobility machinery and classified the flanking regions of ARGs into different MGE types, including ICEs (n = 3), putative ICEs (n = 24), IMEs (n = 6), and other putative conjugative MGEs (n = 22). Nearly half of the identified ARGs were associated with these chromosomal MGEs, highlighting their diversity and potential role in the horizontal transfer and persistence of ARGs within the swine microbiome.

Chromosomal MGEs were classified based on hallmark genes associated with bacterial mobility, including excision/integration, mobilization, and conjugation. Among excision/integration genes, the most prevalent across swine isolates were tyrosine integrases (*PF00589*: phage integrase; identified nearby 16 ARGs), resolvases (*PF00239*; identified nearby 15 ARGs), and serine integrases (*PF07508*: site-specific recombinases; identified nearby 6 ARGs). We also identified a variety of genes within the relaxase protein family (*PF03432*), including those coding DNA mobilization (*MOB*) proteins such as *MOB*_*V*_ (n = 8), and *MOB*_*F*_ (n = 6). The *VirB4* protein, essential for conjugative MGEs, was identified near 24 ARGs across different swine isolates. Of the eight known mating pair formation (MPF) systems [46], six were detected, including MPF types B, F, G, I, FA, along with the predominant FATA subclasses *cd411, gbs1354, prgC*, *prgF*, and *prgK*.

### Distribution of ARGs across extrachromosomal MGEs

In addition to the search for MGEs in draft isolate genomes, our plasmid-focused assembly of sequencing reads using plasmidSpades revealed the presence of 57 circular contigs of varying sizes across swine isolates, ranging from 1,675 to 137,933 bp (average size 13,483bp, ± 19,209). Of these, 21 were recovered from the sequencing reads of *L. reuteri* isolates, 9 from *Lactobacillus johnsonii* isolates, and 7 from *S. alactolyticus* (Additional File 4). Among these, two circular contigs originating from PI_211 and PI_373 (both *L. reuteri*) were classified as dsDNA phages by virsorter2. Fourteen circular contigs exhibited high similarity to known plasmid sequences in the PLSDB database [42], with BLASTn matches showing > 95% identity and > 80% query coverage. Notably, circular contigs from *S. alactolyticus* isolates PI_176 and PI_317 were identical to the *Enterococcus faecium* plasmid *pNUITMV13_2*. Additionally, a circular contig from *Catenibacterium mitsuokai* (PI_167) matched the *Salmonella enterica* plasmid *pN18S0406-4*, while a contig from *Streptococcus orisratti* (PI_182) was identical to the *Streptococcus parasuis* plasmid pSUT-380-2 (Additional File 6). Despite the relatively low number of plasmid and phage sequences identified by the aforementioned tools, comprehensive annotation of all circular contigs using the Pfam database revealed the widespread presence of genes encoding replication proteins (e.g., *repA, repB, repC*), mobilization proteins (e.g., *MobV, MobQ, MobC*), and other plasmid- and phage-associated genes, including replication initiation proteins and site-specific DNA recombinases, across nearly all contigs (see Additional File 5 for detailed gene annotation of circular contigs). Moreover, the search for ARGs in circular contigs identified several genes conferring resistance to lincosamides (*lnuA* and *lnuB* in *L. reuteri* and *S. alactolyticus* isolates), aminoglycosides (*ANT(6)-Ia* in *S. alactolyticus*), tetracyclines (*tetO* in *S. alactolyticus*), and pleuromutilin-lincosamide-streptogramin resistance (*lsaE* in two *S. alactolyticus* isolates; Additional File 7).

### Distribution of the identified ARG-MGE complexes in public swine microbiota databases

**Fig. 4:**
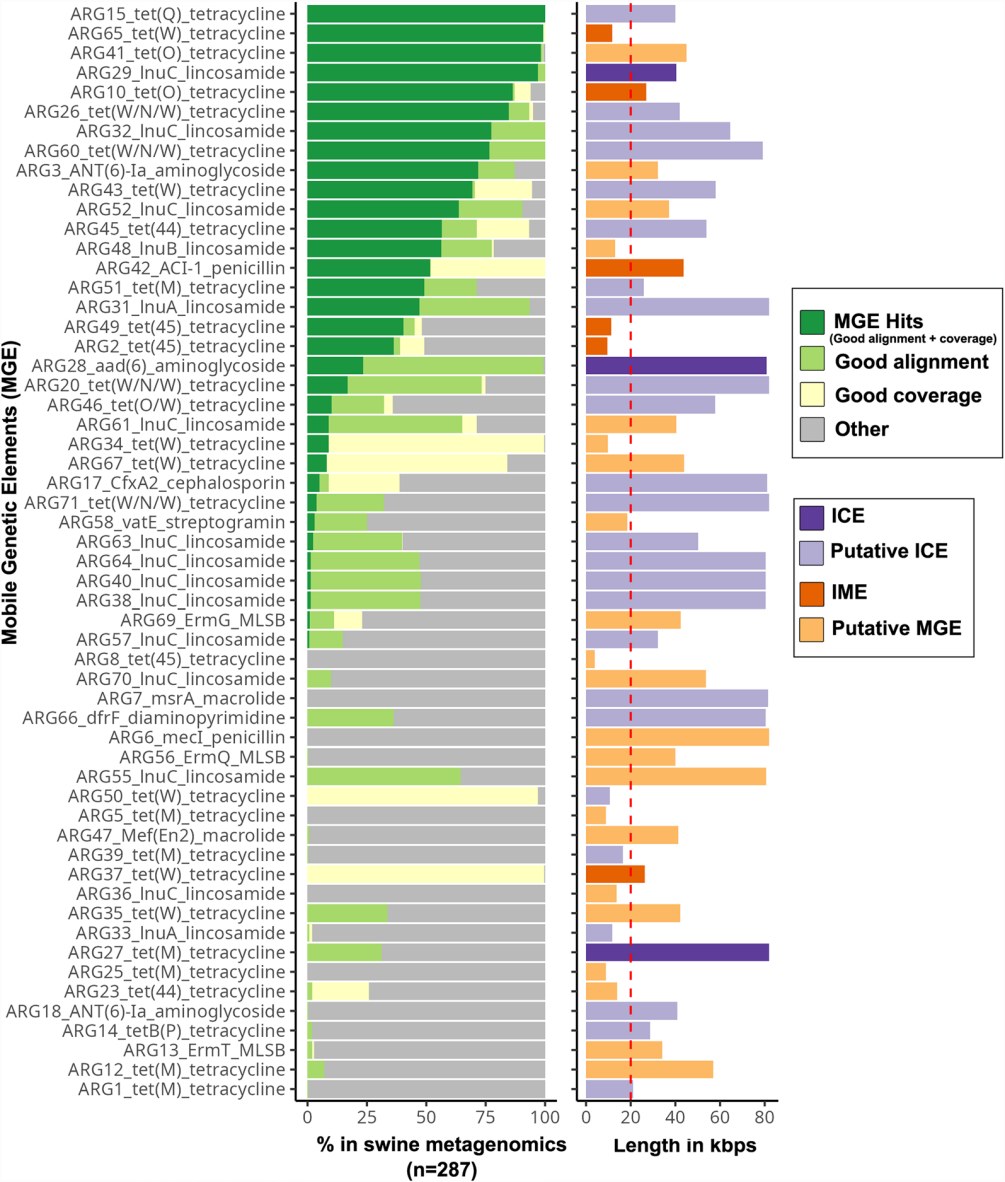
Distribution of the identified ARG-associated MGEs in public swine metagenomic datasets. Metagenomics reads from the fecal microbiome of 287 pigs across three countries (France (n = 100), Denmark (n = 100) and China (n = 87)) were mapped to the retrieved fraction of the contigs containing ARGs and signature genes of mobility machinery. Samples were considered to contain the target ARG-associated MGEs if their metagenomes had “good alignment” (defined by greater than 80% coverage of each ARG-MGE sequence or more than 20,000 bp coverage of the extracted ARG-MGE complexes) and “good coverage” (defined by a minimum of 10 × average base coverage across the aligned region). The bar chart on the left indicates the percentage of swine metagenomes with good alignment and coverage (dark green), good alignment but low coverage (light green), good coverage but short alignment region (yellow), and those with poor coverage and alignment (light grey). The bar chart on the right shows the sequence length of the ARG-associate MGEs identified in the present study, colour coded according to MGE classifications

Finally, to further assess the presence and distribution of homologous sequences to the identified ARG-associated MGEs in the gut microbiome of other swine populations, metagenomic sequencing reads from a large-scale swine microbiome dataset [45] representing the fecal microbiome of pigs from France (n = 100), Denmark (n = 100) and China (n = 87) were mapped to the retrieved fraction of the contigs containing ARGs and signature genes of mobility machinery identified in Fig. 3. We observed that of the 55 chromosomal MGE-associated ARGs identified in our dataset, 18 were detected with high sequence coverage and identity in the fecal microbiome of 50% or more of the abovementioned pig population (Fig. 4). Notably, ten of these highly conserved MGE-associated ARGs carried genes predicted to confer tetracycline resistance, and five carrying ARGs associated with lincosamide resistance. In addition, read mapping of the metagenomic dataset against the 57 circular contigs identified from swine isolates revealed eleven highly conserved putative plasmid sequences present across the studied pig population. These included five circular contigs found in *S. alactolyticus* isolates, two in *Mediterraneibacter faecis* isolates, one in *Coprococcus catus*, one in *Fusicatenibacter saccharivorans*, and one in *L. reuteri*, which all had high sequence coverage and identity in over 40% of the metagenomics samples. Interestingly, a few of these conserved circular contigs harbored ARGs, including contigs from *S. alactolyticus* isolates PI_317, PI_176, PI_188 and PI_199 carrying ARGs conferring resistance to aminoglycosides, lincosamides and tetracyclines (Supplemental Fig. 1 and Additional File 7).

## Discussion

By employing anaerobic culturing techniques and WGS, we created a biobank and high-resolution genome database of isolates spanning several major bacterial families and genera of the swine gut microbiota in this study. Our database revealed a notable prevalence of ARGs and MGEs in isolates from pigs reared under conditions with no documented exposure to antimicrobials. These genes may have been acquired vertically from the dam, from the environment, or due to intrinsic resistance mechanisms inherent in bacterial populations [4, 47].

The findings of our study align closely with the pioneering work of Chen et al*.* [18] who constructed a comprehensive gene catalog, along with the MAGs of the swine gut microbiome. Similar to our findings, the authors identified tetracycline, lincosamide and aminoglycoside resistance to be among the most prevalent ARGs in the swine gut microbiome. However, due to inherent limitations of metagenome MAGs, such as chimerism and genome fragmentation, this approach often fails to assemble repeat elements, including the 16S rRNA gene and transposons, in microbial genomes. As a result, it has a limited ability to accurately identify the bacterial host range and the association of ARGs with MGEs [20, 21]. Our isolate based WGS approach enabled us to overcome these limitations, providing more precise profiling of ARGs in the context of their bacterial host range and association with MGEs. Additionally, by having access to viable isolates we were able to perform phenotypic AST to verify whether genotypic resistance corresponds to phenotypic expression. 

Similar to our findings, Munk et al. [48] previously reported tetracycline resistance as the most abundant ARGs from pig metagenomes. This is likely due to the widespread and prolonged application of tetracyclines within swine production systems [49]. Alongside tetracyclines, lincosamides have historically been widely used in swine farming, particularly in finishing pigs, to prevent and treat enteric diseases [50]. This may explain the high prevalence of lincosamide resistance genes, which followed tetracycline resistance genes in our study. Another study by Mencía-Ares et al. [8] employed WGS to detect ARGs and MGEs in 466 *Escherichia coli*, *Enterococcus*, *Campylobacter*, and *Staphylococcus* species isolated from swine farms, confirming tetracycline resistance as the most prevalent resistance type. However, their analysis was limited by its focus on a narrow range of bacterial species and reliance on reference-based detection of MGEs which limits the ability of detecting novel classes of MGEs in less studied lineages of commensal bacteria. In contrast, our study leveraged the use of pHMM to identify signature genes associated with mobility machinery in microbial chromosomes. This approach enabled us to identify prevalent MGEs, including ICEs, IMEs, and phages across commensal lineages of the swine gut microbiota.

Although our isolate collection is limited in terms of geographical location, sample size, and species and strain diversity, it aligns closely with phylogenetic diversity trends reported in other major studies of the swine gut microbiome [18, 45, 51]. Among the most prevalent families in our collection were *Lactobacillaceae, Streptococcaceae,* and *Lachnospiraceae*. The high abundance of *Lactobacillaceae* and *Lachnospiraceae* families in both culture-based [51, 52] and sequenced based [53–55] studies of the swine gut microbiota likely reflect their important functional roles within the gut ecosystem. For instance, species within *Lactobacillaceae* and *Lachnospiraceae* are well-known for their ability to adhere to the intestinal mucosa [56], produce antimicrobial peptides [57] and short chain fatty acids [58], and regulate immune homeostasis [57, 59].

However, several isolates from our collection have been identified in previous studies as opportunistic and zoonotic pathogens. For example, *Streptococcus suis*, a species we isolated, is well-documented for causing respiratory infections and bacterial meningitis in swine [60, 61]. *S. suis* has been linked to significant economic losses in swine farms, particularly during the nursery phase. A European study reported that approximately 3.3–4% of pigs in this phase were affected by *S. suis* infections [62]. Similarly, *S. hominis*, another of our isolates, has been linked to severe human infections, including endocarditis, bacteremia, and endophthalmitis [63]. Additionally, we cultured *S. alactolyticus*, which, while typically regarded as commensal, has been associated with cases of human endocarditis [64, 65]. We found association of these isolates with several ARGs, which underscore their potential risk to veterinary and public health since horizontal transfer of ARGs can occur more readily among closely related microbes within the same genus [66].

Another important observation of our study was the high association of ARGs with MGEs, including ICEs, plasmids, and phages. These MGEs facilitate HGT and likely contribute to the dissemination of resistance within microbial ecosystems [67]. Of particular concern was the identification of putative plasmids from the commensal *S. alactolyticus* that closely resembled a plasmid from *Enterococcus faecium*. *E. faecium* is a common commensal in the gastrointestinal tracts of both humans and animals, belonging to the *Enterococcus* genus, whose members are typically found at concentrations ranging from 10^3^ to 10⁷ CFU per gram of feces [68]. However, despite its presence in the gut microbiota of healthy animals, certain strains of *E. faecium* has been classified by the World Health Organization as high-priority multidrug-resistant pathogens, particularly those carrying vancomycin resistance, and are frequently implicated in central line-associated bloodstream infections [69]. In addition to the nosocomial infections, *E. faecium* can cause urinary tract infections, endocarditis, abdominal and biliary tract infections, and wound infections [68]. The putative plasmids identified in *S. alactolyticus* are particularly concerning, as they carry multiple ARGs, including those conferring resistance to tetracyclines. This is especially troubling given that certain tetracycline resistance genes have been implicated in reduced susceptibility to tigecycline, one of the last-resort antibiotics used to treat infections caused by vancomycin-resistant Enterococcus (VRE) [70].

The detection of putative plasmids in *Streptococcus orisratti* and *Catenibacterium mitsuokai*, which showed high sequence similarity with plasmids from *Streptococcus parasuis* and *Salmonella enterica*, respectively, underscores the potential for MGE dissemination across diverse bacterial taxa. Given that *S. parasuis* is a zoonotic opportunist [71] and *S. enterica* is a major foodborne pathogen affecting both humans and animals [72], the presence of such contigs in gut commensals raises concerns about the potential horizontal transfer of ARGs to pathogenic species. This is particularly significant as gene transfer has been documented not only across genera but even across phyla. While our study does not experimentally confirm interspecies transmissibility of the identified putative plasmids, their presence highlights the need for further mechanistic investigations into the genetic exchange between commensals and zoonotic pathogens, especially in the context of the AMR footprint of livestock production systems.

Furthermore, the presence of ICEs and putative ICEs adjacent to ARGs, such as *tetWNW* and *tetO*, highlighted the potential role of these MGEs in their mobility. ICEs are self-transmissible, diverse genetic elements with three core modules (conjugation, recombination, and regulation) clustered together [73, 74]. They carry functional genes like virulence factors, or ARGs as accessory components alongside their core modules. They are usually transferred as linear single-stranded DNAs in most bacteria (except *Streptomyces*, which uses a double-stranded DNA approach), the transfer being dependent on relaxase [74, 75]. We detected several relaxase genes surrounding ARGs, including families of canonical relaxases (*MOB*_*C*_*, MOB*_*F*_*, MOB*_*H*_*, MOBP*_*1*_*, MOB*_*Q*_*,* and *MOB*_*V*_*),* as well as non-canonical relaxase (*MOB*_*T*_), which are essential in the transfer of ssDNA in the conjugation process [75]. Notably, approximately 25% of the MGE-associated ARGs identified in our study were also detected in the fecal microbiome dataset generated by Xiao et al. [45] from pig populations in France, Denmark, and China. While metagenomic read mapping cannot confirm a definitive linkage between the target ARGs and MGEs or resolve the microbial host range of these complexes, the substantial coverage of these genomic regions across the recruited metagenomic datasets suggests a high prevalence and stability of the studied ARGs and MGEs within the healthy swine gut microbiome. This finding highlights the potential threat posed by the dissemination of the swine AMR repertoire via feces to the broader environment.

Interestingly, despite the high frequency of ARGs, there were discrepancies between genotypes and phenotypes in several isolates, such as *S. hominis* and *M. elsdenii*, where resistance genotypes did not translate into phenotypic resistance. Whereas, *Staphylococcus, Coprococcus,* and *Mitsuokella* isolates showed AMR phenotypes without having the genotype. Such lack of correspondence between AMR genotype and phenotype is often reported in genomic studies [76, 77]. This could be attributed to different factors, such as the presence of uncharacterized ARGs, overexpression of efflux pumps, intrinsic resistance mechanisms, drug indifference, or the growth in biofilms [78]. This illustrates the complexity of AMR expression in natural microbiomes and shows the need for integrating phenotypic and genomic analyses to better understand the dynamics of AMR and its mechanisms in microbial ecosystems.

## Conclusion

Overall, our study offers novel insights into the lineage-specific distribution of ARGs and their association with MGEs within the swine gut microbiome. We identified several commensal microbes as carriers of clinically relevant ARGs, even in the absence of prior antimicrobial exposure in their host animals. Notably, some of these microbes are considered to be zoonotic pathogens capable of infecting both pigs and humans. Tetracycline and lincosamide resistance genes emerged as the most prevalent ARG classes, despite ongoing efforts to restrict prophylactic use of these antimicrobial in pig production. Importantly, we found that the majority of ARGs were linked to MGEs, including ICEs, plasmids, and phages, suggesting their potential for horizontal transfer. Several putative plasmids identified in commensal isolates shared high sequence similarity with plasmids from pathogens of both human and veterinary importance, highlighting the risk of ARG dissemination not only among gut commensals but also to opportunistic environmental and zoonotic pathogens. These findings underscore the critical need for microbiome-informed AMR surveillance and responsible antimicrobial stewardship in livestock systems. The genomic dataset generated in this study provides a valuable resource for tracking resistance genes within the agri-food chain and supports the development of targeted strategies to curb the spread of antimicrobial resistance.

## Supplementary Information

Additional file1 (XLSX 30 KB)

Additional file2 (XLSX 51 KB)

Additional file3 (XLSX 13 KB)

Additional file4 (XLSX 11 KB)

Additional file5 (XLSX 66 KB)

Additional file6 (XLSX 12 KB)

Additional file7 (XLSX 16 KB)

Additional file8 (PDF 582 KB)

## Data Availability

The sequencing data (Illumina reads) and assembled genomes were deposited into the Sequence Read Archive (SRA) of NCBI (http://www.ncbi.nlm.nih.gov/sra) and can be accessed via BioProject number PRJNA1187550.
