# Supplementary material for: Genomic characterization of antimicrobial resistance and mobile genetic elements in swine gut bacteria isolated from a Canadian research farm
Source: Anim Microbiome. 2025 Jun 18;7:66. doi: 10.1186/s42523-025-00432-w (PMC12175345; doi:10.1186/s42523-025-00432-w)
Supplement: Supplementary file 8 — Additional file8 (PDF 582 KB) [file 42523_2025_432_MOESM8_ESM.pdf]

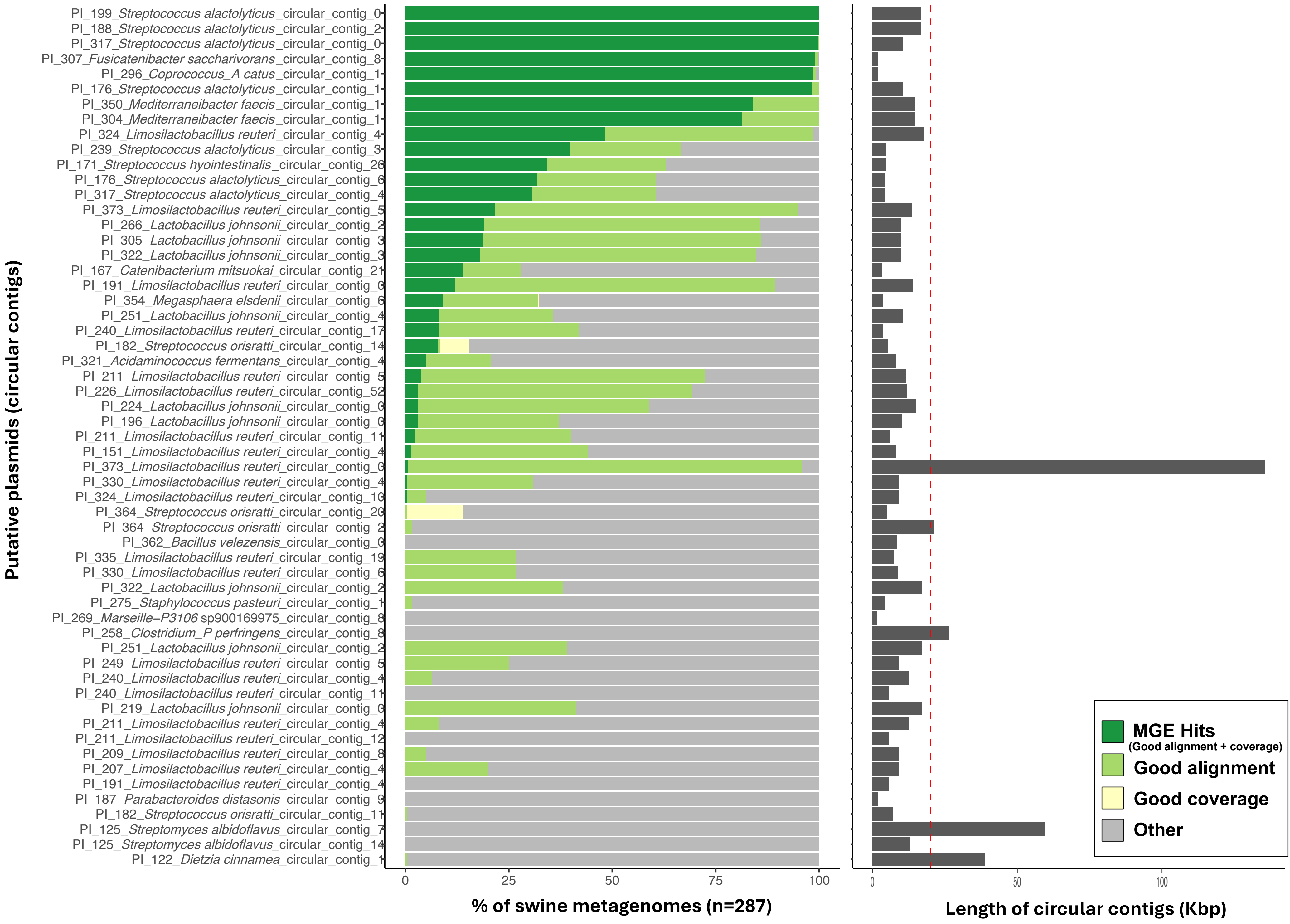

Supplemental Fig 1: Distribution of the identified putative plasmids in public swine metagenomic datasets. Metagenomics reads from the fecal microbiome of 287 pigs across three countries (France (n=100), Denmark (n=100) and China (n=87)) were mapped to circular contigs assembled from the sequencing reads obtained from each isolate. Metagenomic samples were considered to have a high-coverage of the identified putative plasmids if they had "good alignment" (defined by greater than 80% coverage of each putative contig sequence) and "good coverage" (defined by a minimum of 5x average base coverage across the aligned region). The bar chart on the left indicates the percentage of swine metagenomes with good alignment and coverage (dark green), good alignment but low coverage (light green), good coverage but short alignment region (yellow), and those with poor coverage and alignment (light grey). The bar chart on the right shows the sequence length of the putative plasmids.
